# Classifying and clustering mood disorder patients using smartphone data from a feasibility study

**DOI:** 10.1038/s41746-023-00977-7

**Published:** 2023-12-21

**Authors:** Carsten Langholm, Scott Breitinger, Lucy Gray, Fernando Goes, Alex Walker, Ashley Xiong, Cindy Stopel, Peter Zandi, Mark A. Frye, John Torous

**Affiliations:** 1 Division of Digital Psychiatry, Beth Israel Deaconess Medical Center, Boston, MA 02115 USA; 2 Department of Psychiatry & Psychology, Mayo Clinic, Rochester, MN 55902 USA; 3 Department of Psychiatry and Behavioral Sciences, Johns Hopkins School of Medicine, Baltimore, MD 21218 USA

**Keywords:** Biomarkers, Depression

## Abstract

Differentiating between bipolar disorder and major depressive disorder can be challenging for clinicians. The diagnostic process might benefit from new ways of monitoring the phenotypes of these disorders. Smartphone data might offer insight in this regard. Today, smartphones collect dense, multimodal data from which behavioral metrics can be derived. Distinct patterns in these metrics have the potential to differentiate the two conditions. To examine the feasibility of smartphone-based phenotyping, two study sites (Mayo Clinic, Johns Hopkins University) recruited patients with bipolar I disorder (BPI), bipolar II disorder (BPII), major depressive disorder (MDD), and undiagnosed controls for a 12-week observational study. On their smartphones, study participants used a digital phenotyping app (mindLAMP) for data collection. While in use, mindLAMP gathered real-time geolocation, accelerometer, and screen-state (on/off) data. mindLAMP was also used for EMA delivery. MindLAMP data was then used as input variables in binary classification, three-group k-nearest neighbors (KNN) classification, and k-means clustering. The best-performing binary classification model was able to classify patients as control or non-control with an AUC of 0.91 (random forest). The model that performed best at classifying patients as having MDD or bipolar I/II had an AUC of 0.62 (logistic regression). The k-means clustering model had a silhouette score of 0.46 and an ARI of 0.27. Results support the potential for digital phenotyping methods to cluster depression, bipolar disorder, and healthy controls. However, due to inconsistencies in accuracy, more data streams are required before these methods can be applied to clinical practice.

## Introduction

Clinical overlap in symptoms between bipolar disorder and depressive disorder complicates accurate diagnosis and personalized treatment. Cross-sectionally, unipolar depressive symptoms may often be difficult to distinguish from bipolar depressive symptoms^1^. For patients presenting with a first-episode mood diagnosis, ruling out bipolar disorder can be difficult, and recent research suggests that up to 20% of those diagnosed with a depressive disorder may have an underlying bipolar disorder^2^. The diagnostic distinction between unipolar and bipolar depression has clinical implications because first-line treatments (antidepressants, mood stabilizers) are fundamentally different^3^.

With current capabilities, distinguishing unipolar depression from bipolar depression requires careful longitudinal observation. These diagnostic efforts can be burdensome. Therefore, research efforts have emerged with the goal of discovering earlier clinical biomarkers. However, these clinical biomarkers have had limited success in the diagnostic separation of bipolar disorder and major depression^4,5^. In response, there have been numerous calls for new “neurocognitive models of mental illness … that can deliver on substantially richer, multivariate data sets and larger samples than are feasible in the traditional small, single-site studies that dominate the field^6^”.

There has been rising interest in digital biomarkers, especially those involving smartphone data. Smartphone data, because of low costs to implementation and widespread use, has scalable potential. Prior research, described as “digital phenotyping,” involves smartphone-derived markers of human behavior. These markers include sleep duration, screen usage time, geolocation activity patterns, social interactions, and many others. Digital phenotyping has shown feasibility in characterizing both depression and bipolar disorder. For example, recent work has shown that smartphone self-monitored mood is positively correlated with smartphone-measured step count. In addition, the number of smartphone-measured outgoing phone calls is positively correlated with the Young Mania Rating Scales (YMRS) in people with bipolar disorder^7,8^. Other papers have used different smartphone signals, such as geolocation and mobility patterns to predict both the Hamilton Depression Rating Scale (HAM-D) and the YMRS^6^. A 2022 review found 118 articles showing associations between digital phenotyping and depression severity, negative affect, physical activity, social functioning, and sleep quality variability^9^.

Despite the potential for digital methods to phenotype unipolar depression and bipolar disorder, many studies lack of reproducibility or report contradictory results^10,11^. A lack of comparative studies for digital phenotyping in these disorders has been cited as the chief challenge for assessing the validity and reliability of this approach. In addition, to our knowledge, no study has focused on using this method specifically to distinguish between or stratify unipolar depression and bipolar disorder.

Thus, in this paper, we present the results of a digital phenotyping study designed to stratify the digital traces of patients with either unipolar depression, bipolar disorder, or neither. We asked patients to collect smartphone data and answer surveys on a regular basis. Later, we attempt to classify and cluster patients according to their digital data. In our study design, we emphasize reproducibility. To that end, we use an open-source digital phenotyping platform currently employed in the international NIH’s Accelerating Medicine Partnership Schizophrenia Study^12^. We also provide the code used to perform all analyses. To improve reliability, we run the protocol at two unique sites serving patients with differing severity of illness.

## Results

### Binary classification

For each classification performed in this study, multiple models were implemented. We tested and tuned all models three separate times, using different subsets of input variables (active data only, passive data only, or all data). The performances of every best-performing model (highest accuracy on the validation set) for binary classifications were compiled and reported in Table 1.

**Table 1:** Binary classification results.

Classification type	Best model	Validation accuracy	Test accuracy	Test AUC	Features
Control vs non-control	Random Forest	88.9	84.6	0.91	All
Bipolar vs unipolar	SVM	59.1	57.1	0.61	All
Control vs non-control	Naive Bayes	79.3	84.6	0.93	Active
Bipolar vs unipolar	Logistic Regression	66.7	64.3	0.62	Active
Control vs non-control	Random Forest	72.6	69.2	0.65	Passive
Bipolar vs unipolar	Decision Trees	66.7	50	0.52	Passive

AUC was strong (AUC > 0.9) when using all data or only active data to classify patients as control or non-control. Using passive data alone provided moderate predictive value (AUC = 0.65). Classifying patients according to diagnosis among the non-control samples was moderately successful when using all data or active data, but passive data alone was not able to distinguish between non-control groups (AUC = 0.52).

### KNN classification

Three-group k-nearest neighbors classification was performed to determine whether the data could predict to which of the three diagnosis groups each participant belonged. Results were quantified and compiled in Table 2.

**Table 2:** Three-group KNN classification results presented as the percent of participants in each diagnosis group who were correctly identified by the model.

Diagnosis group	All features	Active features	Passive features
Control accuracy	91%	91%	55%
MDD accuracy	30%	0%	50%
Bipolar accuracy	50%	83%	39%
Overall accuracy	56%	64%	46%

The KNN model appears to classify participants into control or non-control moderately accurately. Although using passive data alone produced lower accuracy when classifying patients as control or non-control, this accuracy still greatly outperformed random guessing. There was lower accuracy associated with classifying participants as part of the MDD or bipolar groups. Interestingly, when using only active features (surveys) as predictor variables, the model had the highest overall accuracy with high control accuracy. However, the model appeared to classify nearly all non-control participants as belonging to the bipolar group, explaining the zero MDD accuracy. On the contrary, when using only passive data the model displayed a lower overall accuracy but managed to distinguish some participants between non-control groups.

### Clustering

**Fig. 1:**
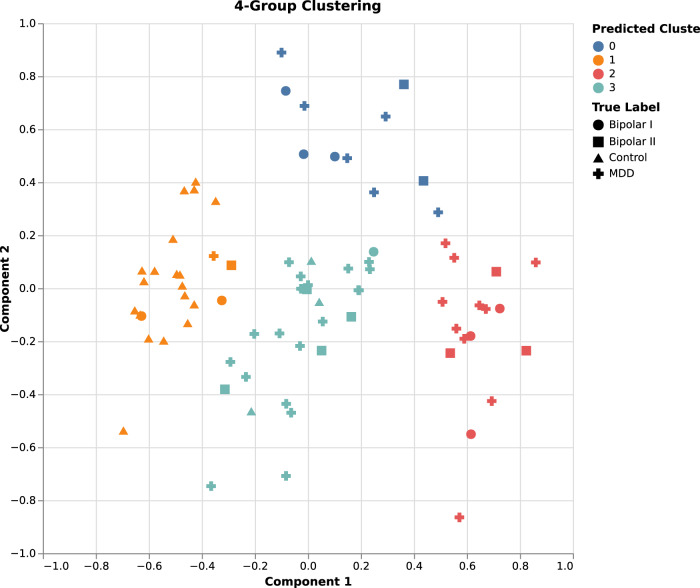
K-means clustering results. Distinct clusters are represented by the blue, orange, magenta, and turquoise colors. Patient diagnosis labels are represented by circle, square, triangle, and plus symbols.

K-means clustering was performed on all data using mean imputation. Clustering results, broken into principal components, were compiled into Fig. 1.

Performing k-means clustering yielded a silhouette score of 0.46 and an ARI of 0.27 (Fig. 1).

## Discussion

This study investigated using passively collected smartphone digital phenotyping data to predict the diagnosis of mood disorder patients. We found the best-performing binary classification models to have moderate to high success. We also showed that k-means clustering algorithms using *K* = 4 had some success stratifying patients according to silhouette scores. These results suggest that this approach is feasible for the stratification of MDD and bipolar disorder, though inconsistent classification accuracy warrants further investigation with larger sample sizes and more data streams.

Digital phenotyping methods involve both active assessments, via surveys, and passive assessments, via sensors. Using passive data alone as a predictor variable had moderate success (AUC = 0.65) when classifying participants as control or non-control. Although the ability to determine whether a participant can be classified as control or non-control has value in showcasing how smartphone data can produce a digital biomarker of clinical conditions, the true potential of classification comes in classifying patients according to their non-control clinical diagnosis. Doing so not only has clinical utility as a diagnostic tool but can also help elucidate the relationships between diagnosis, symptoms, and the features comprising a digital phenotype. However, classifying patients according to their non-control diagnosis had less success using passive data alone (AUC = 0.52). When introducing survey data, AUC improved only slightly (AUC = 0.62).

Although passive data alone cannot be relied upon to drive predictive methods in clinical care at this time, these results show promise nonetheless. The ease, lack of bias, and scalability of smartphone data collection means it can be broadly implemented with little cost. Because of this convenience, any AUC score showing it has moderate predictive value on its own shows great potential. These results warrant additional investigation with additional data streams and larger sample populations to further evaluate and improve upon the value of smartphone-based passive data.

Clustering patient data has perhaps even more potential than classification. Silhouette scores show the ability to stratify patients into partially distinct classes according to their digital data. Low ARI scores, however, suggest the stratification between these clusters does not consistently agree with clinical diagnoses. Although this could be a product of inconsistent data, this finding could also be explained by imprecise symptomatic labeling.

These results have a strength in reproducibility, transparency, and feasibility. While clustering and classification studies are increasingly common today, they are often difficult to scale and challenging to replicate. On the contrary, our approach is designed for replication. None of the features used as input variables require additional equipment like wearable devices. Costs are minimal to research groups, allowing for easy validation.

These results suggest there is potential for new research exploring this approach with larger samples across more sites. But these results also offer practical implications today. Digital phenotyping data, although today unable to drive care alone, can be used to augment clinical care for patients with mood disorders and help people better understand the relationships between their own behaviors and symptoms^13^.

Limitations in this study suggest possibilities for future research. We only explored a small number of measurable data vectors, and given the proliferation of biometric sensors, there remain further opportunities to engage with higher dimensional data collection. Future research could not only validate these results but also have greater success by including more features and implementing protocols to reduce missingness. The reliability of these classification models in real life is limited by the reliability of the gold-standard (clinician diagnosis). However, this only emphasizes the importance of new diagnostic methods. Other limitations include combining Bipolar I and Bipolar II patients into one group and performing data imputation. If clustering methods continue to improve and can potentially stratify patients with more precision than symptom-based clinical diagnosis, future research should be centered around the potential for whether digital phenotyping clusters can better predict medication response and whether clusters or biotypes align across new predictor variables and study sites^14^. Lastly, these results should be validated in future studies with larger sample sizes to determine the degree of overfitting associated with these results.

Overall, this study showed the potential for digital phenotyping in classifying patients according to their diagnosis, although results are at risk of overfitting due to the small sample size. Results could be weakened by overlap between clinical diagnoses, suggesting unsupervised clustering could have greater potential in labeling patients. The ability to precisely cluster patients could lead to greater precision in labeling patients and therefore could be useful in medication assignment. These results should be replicated and validated, which can be easily accomplished due to the open-source nature of the tools used and the analysis pipeline.

## Methods

### Recruitment and protocol

Each of the study sites received approval by its institutional review board (Mayo Clinic: IRB 20-008773, Johns Hopkins University: IRB 00285631), and every participant provided written informed consent. The study protocol, not previously reported, is described in a separate paper currently in review. Study recruitment and protocol were identical between both study sites in terms of smartphone data collection. Participant demographics are summarized in Table 3.

**Table 3:** Participant demographics summary.

Gender	Number	Percent
F	84	72
M	32	28
Diagnosis		
Control	35	30
MDD	53	46
Bipolar I	13	13
Bipolar II	15	11
Race		
White	81	70
Asian	15	13
Black	11	9
Hispanic	2	2
Asian/White	2	2
Black/White	1	1
American Indian	1	1
Other	3	2

### Diagnostic criteria

Participants had their diagnosis confirmed using the Mini International Neuropsychiatric Interview. Depressive symptom severity was assessed using the Quick Inventory of Depressive Symptomatology—Clinician Rating. The degree of hypomania/mania was assessed using the Young Mania Rating Scale. Rating of videotaped patient interviews, according to the QIDS and YMRS, was performed by six raters, including two psychiatrists. Inter-rater reliability was found to be 0.92 (QIDS) and 0.96 (YMRS).

### Surveys

Participants responded to surveys using mindLAMP, an open-source mental health intervention and monitoring application^15^ over the course of 12 weeks in 2021-2023, depending on the participant’s start date. mindLAMP sent notifications three times per week to participants, prompting them to respond to in-app surveys measuring self-reported depression (PHQ-2)^16^ and anxiety (GAD-2)^17^. Survey scores were mapped to integer values (0-6). Participants responded to a mean of 33 total surveys each.

### Sensor data

Mobile devices contain sensors which passively record information about user activity, including but not limited to geolocation, motion, exercise, device rotation, and many others. While participants had mindLAMP installed on their devices, mindLAMP passively collected smartphone sensor data and sent this data to secure servers. This sensor data (“passive data”) was then processed into more meaningful metrics for analysis. To collect passive data, participants had to enable data collection permissions for mindLAMP in iOS or Android settings.

### Data processing

mindLAMP participant data was stored in secure AWS servers and obtained for analysis using the HIPAA-compliant LAMP API^15^. Raw passive data was processed and converted to more interpretable metrics (“features”) using the LAMP-cortex data analysis Python package developed by the Division of Digital Psychiatry at Beth Israel Deaconess Medical Center (BIDMC)^15^. For this study, sensor data was processed into the following features: home time (time spent at home), entropy (a quantified measure of how often a patient changed their location), sleep duration, and screen duration (time spent using device). Sleep duration was estimated from periods of smartphone inactivity and was processed from device acceleration and screen usage data.

For each participant, all data was split into 24-h intervals starting from the timestamp of the first survey taken to the timestamp of the last survey taken. Survey results for each participant were averaged over each interval (e.g., if two mood surveys were taken during the same 24-h period, their scores were averaged) and the derived features for each participant were summed over the interval (e.g., we summed the total number of hours the participant spent at home over each 24-h period).

For every 24-h bin, we also calculated passive data quality. We define data quality as the percent of 1-h bins that contain at least one GPS data point. We temporally aligned per-interval data quality with all other data streams and filtered for data frequency, excluding bins with data quality below 0.8, under the assumption that low-quality bins would produce biased results. Afterward, the means and sample standard deviations of all survey scores and features were calculated over all high-quality 24-h bins per participant. These means and variances were later used as predictor variables in regression models.

A number of participants failed to obtain a sufficient number of samples for calculating mean and variance of their survey and feature data, producing missing data. A summary of the amount of missing data (after filtering for data quality) is provided in Table 4. Because of the number of input variables used in the regression models, excluding participants with any amount of missing data would drastically reduce the number of participants with applicable data. Instead, we imputed missing values using mean feature values.

**Table 4:** Percent missing data by feature variable type.

Features	Percent of data missing
All	13.80%
Active only	11.20%
Passive only	14.90%

### Classification

Participants were classified in three ways using the scikit-learn package (Python 3). Due to low sample size, for the purpose of these predictions, we combined the participants diagnosed with BPI or BPII into the same group (“bipolar group”). First, we used standard predictive methods to determine whether the available data was sufficient to predict if participants belonged to the control group or to one of the non-control groups (MDD, bipolar group). Second, we predicted whether non-control patients belonged to the MDD group or the bipolar group. Third, we used a three-group KNN classification model to predict to which group each participant belonged (control, MDD, or bipolar group). For binary classification, a variety of model types were tested: Logistic Regression (LR), Support Vector Machines (SVM), Decision Trees (DT), Random Forest (RF), Naive Bayes (NB), and K-Nearest Neighbors (KNN). All tested binary models were tuned for hyperparameters via k-fold (*k* = 5) cross-validation using the GridSearchCV module from the scikit-learn package to maximize the accuracy of each model. The data was split into a training set (50%) and testing set (50%). After hyperparameter tuning on the training set, the model was trained using best-performing hyperparameters on the entire training set and tested on the independent testing set. The three classification types are summarized in Table 5. In this paper, for each of the three classification types listed, we present the testing set AUC/accuracy of the model with the best validation set accuracy.

**Table 5:** Classification type overview.

Comparison groups	Model	Cross-validation	Evaluation criteria
Control vs non-control	LR, SVM, DT, RF, NB, KNN	Repeated (*n* = 3) Stratified K-fold (*k* = 5)	Testing set AUC
Bipolar depression vs unipolar depression	LR, SVM, DT, RF, NB, KNN	Repeated (*n* = 3) Stratified K-fold (*k* = 5)	Testing set AUC
Control vs bipolar depression vs unipolar depression	KNN (*K* = 3)	Repeated (*n* = 3) Stratified K-fold (*k* = 5)	Overall accuracy (percent correct)

We also sought to ascertain the relative contribution of the different categories of predictor variables (active data vs passive data). Therefore, we performed each binary classification three times: using only active data as input variables, using only passive data as input variables, and using all data as input variables.

### Clustering

K-means clustering (*K* = 4) was performed using the K-Means module in the scikit-learn package. Data was scaled using the StandardScaler module and principal component analysis was performed using the PCA module to reduce the dimensionality of the predictors. There was no hyperparameter tuning; default sklearn parameters were used (scikit-learn.org/stable/modules/generated/sklearn.cluster.KMeans.html). Clustering results were quantified using silhouette scores and adjusted rand index (ARI) scores.

## Data Availability

The data used in this study contains sensitive patient information and cannot be shared publicly but may be made available upon request.

## Code availability

The code used to produce this analysis is provided at: github.com/BIDMCDigitalPsychiatry/Cluster-Study.
